# Remote Cognitive Screening Of Healthy Older Adults for Primary Care With the MyCog Mobile App: Iterative Design and Usability Evaluation

**DOI:** 10.2196/42416

**Published:** 2023-01-10

**Authors:** Stephanie Ruth Young, Emily Gardiner Lattie, Andrew B L Berry, Lynn Bui, Greg Joseph Byrne, Julia Noelani Yoshino Benavente, Michael Bass, Richard C Gershon, Michael S Wolf, Cindy J Nowinski

**Affiliations:** 1 Department of Medical Social Sciences Feinberg School of Medicine Northwestern University Chicago, IL United States; 2 Do Dac Studio Seattle, WA United States; 3 Center for Applied Health Research on Aging Feinberg School of Medicine Northwestern University, Chicago, IL United States

**Keywords:** human-centered design, mobile health, mHealth, usability, cognitive screening, older adults, mobile phone

## Abstract

**Background:**

Annual cognitive screening in adults aged >65 years can improve early detection of cognitive impairment, yet less than half of all cases are identified in primary care. Time constraints in primary care settings present a major barrier to routine screening. A remote cognitive screener completed on a patient’s own smartphone before a visit has the potential to save primary care clinics time, encourage broader screening practices, and increase early detection of cognitive decline.

**Objective:**

We described the iterative design and proposed the implementation of a remote cognitive screening app, MyCog Mobile, to be completed on a patient’s smartphone before an annual wellness visit. The research questions were as follows: What would motivate primary care clinicians and clinic administrators to implement a remote cognitive screening process? How might we design a remote cognitive screener to fit well with existing primary care workflows? What would motivate an older adult patient to complete a cognitive screener on a smartphone before a primary care visit? How might we optimize the user experience of completing a remote cognitive screener on a smartphone for older adults?

**Methods:**

To address research questions 1 and 2, we conducted individual interviews with clinicians (n=5) and clinic administrators (n=3). We also collaborated with clinic administrators to create user journey maps of their existing and proposed MyCog Mobile workflows. To address research questions 3 and 4, we conducted individual semistructured interviews with cognitively healthy older adults (n=5) and solicited feedback from a community stakeholder panel (n=11). We also tested and refined high-fidelity prototypes of the MyCog Mobile app with the older adult interview participants, who rated the usability on the Simplified System Usability Scale and After-Scenario Questionnaire.

**Results:**

Clinicians and clinic administrators were motivated to adopt a remote cognitive screening process if it saved time in their workflows. Findings from interviews and user journey mapping informed the proposed implementation and core functionality of MyCog Mobile. Older adult participants were motivated to complete cognitive screeners to ensure that they were cognitively healthy and saw additional benefits to remote screening, such as saving time during their visit and privacy. Older adults also identified potential challenges to remote smartphone screening, which informed the user experience design of the MyCog Mobile app. The average rating across prototype versions was 91 (SD 5.18) on the Simplified System Usability Scale and 6.13 (SD 8.40) on the After-Scenario Questionnaire, indicating above-average usability.

**Conclusions:**

Through an iterative, human-centered design process, we developed a viable remote cognitive screening app and proposed an implementation strategy for primary care settings that was optimized for multiple stakeholders. The next steps include validating the cognitive screener in clinical and healthy populations and piloting the finalized app in a community primary care clinic.

## Introduction

### Background

Early detection of cognitive impairment is important for optimal care planning, management of symptoms and comorbidities, and determination of appropriate caregiver involvement [1,2]. For over a decade, Medicare has covered an annual wellness visit for adults aged >65 years (“older adults”) that requires a routine cognitive assessment to detect impairment, yet less than half of cases are identified in primary care settings [3]. Unfortunately, primary care settings face a number of barriers to routine cognitive screening, including lack of time to conduct screenings and limited training and resources available for dementia care [4].

Some studies suggest that computerized cognitive screening tools can help primary care clinicians (PCCs) overcome barriers to screening and improve early detection of impairment [5]. One such computerized screener, MyCog, is a tablet-based app developed for self-administration in a clinical setting. It uses 2 tests from the National Institutes of Health Toolbox Cognition Battery that assess episodic memory (Picture Sequence Memory) and cognitive flexibility (Dimensional Change Card Sorting), both of which have been validated for both research and clinical use [6,7]. Preliminary studies found that MyCog demonstrated good sensitivity and specificity in detecting cognitive impairment when combined with self-reported concern about cognitive problems in clinical populations [8].

Preliminary feedback from PCCs revealed that MyCog saved time but also presented some logistical challenges for primary care clinics, such as room availability and the storage and maintenance of tablet devices [9]. A companion version of the MyCog screener that can be completed remotely before a visit on a patient’s own device has the potential to advance screening practices in primary care by reducing the number of patients who must be screened in person. To be used in clinical practice, a new at-home cognitive screener must be designed and implemented with careful attention paid to the perspectives, motivations, and existing workflows of various stakeholders (ie, clinicians, clinic administrators, and patients).

### Current Cognitive Screening Practices in Primary Care

PCCs have a unique opportunity to track their patients’ cognitive trajectories over time. Routine cognitive screening during primary care visits can promote early diagnosis of neurocognitive disorders; help identify potentially treatable causes of impairment (eg, medication use); enable patient and caregiver education and counseling regarding diagnosis; maximize time for medical, financial, and caregiver support planning; promote management of comorbidities; provide opportunities for research participation; and empower individuals to make critical decisions before impairment becomes severe [10]. Moreover, starting routine cognitive screening while a patient is still cognitively healthy can help establish a baseline level of cognitive functioning to compare against future performance if a decline is suspected [11].

Although memory tasks are the most common, cognitive screeners may also include tests of orientation, executive functioning, verbal fluency, attention, and processing speed [12]. The quality of cognitive screeners varies greatly, but there are several well-validated cognitive screeners that can effectively differentiate healthy aging from pathological decline [13,14]. Studies suggest that most patients in primary care are willing to participate in cognitive screenings [15,16] and perceive at least some benefit to the screening process [15,17]. Importantly, there is some evidence suggesting that screening does not have harmful psychological implications for older adults [18].

Although PCCs generally recognize the benefits of cognitive screening, few PCCs implement cognitive screening best practices [19]. In a survey of PCCs, 41% reported that they administered standardized cognitive screening tests to less than half of their patients with cognitive concerns, and 5% reported never administering these tests at all [20]. There is no nationally recognized standard cognitive screening tool for primary care, and PCCs who administer cognitive screeners tend to use a variety of paper-and-pencil–based assessments at their discretion [21]. The Mini-Mental Status Exam is one of the most popular cognitive screeners among PCCs [21], but several studies have shown that it is neither the most accurate nor the most efficient screener for detecting cognitive impairment [22]. Moreover, errors are common in many paper-and-pencil cognitive screeners, which can further affect their accuracy. A review of 2706 Mini-Mental Status Exam protocols found that almost 24% contained an administration or scoring error [23], and a review of another popular screener, the Mini-Cog, found that 61% of nurses failed to administer or score the test correctly [24].

Well-validated computerized screeners have the potential to improve accuracy and overcome practical obstacles to cognitive screening in primary care settings. Automation of scoring and rote administration tasks can save time and reduce common examiner-related errors [25,26]. In addition to the time-saving benefits, many PCCs appreciate the interpretation guidelines and recommendations offered by computerized screening reports [27], which may help improve approaches to dementia care [5].

Despite their advantages, computerized screeners are not yet commonly used in routine clinical practice [20,28], and there are several potential barriers to their adoption [25]. Some primary care clinics may worry about the acceptability of computerized screeners among their older patients, although research suggests that testing with computers is generally well tolerated by older adults [29]. From a logistical standpoint, most computerized screeners currently administered in clinics are designed for a tablet, laptop, or desktop computer, which the primary care clinic must purchase, maintain, and securely store. The costs of devices and software, training, data security, and technical support issues associated with computerized screeners may present practical obstacles for some clinics [25].

Primary care clinics may also have concerns about how computerized screening tools will integrate with their existing workflows and electronic medical records [30]. In fact, poor fit with existing practices and workflows is one of the most common reasons why implementations of new health care technologies are unsuccessful [31]. Involving clinical end users in the design and development process can help minimize workflow disruptions, maximize usability, and overcome other barriers to the adoption of new technologies in clinical settings [31]. A potential strategy to reduce the impact of cognitive screening on the clinical workflow is to give patients the opportunity to complete screeners on their own before their in-person visits.

### At-home Cognitive Screening

At-home cognitive screening is a new and promising concept that may help further improve the efficiency of computerized assessment [28]. Depending on their current screening practices and tools, clinics could save up to 30 minutes per patient if patients complete a cognitive screener before the visit [32]. Moreover, PCCs have the potential to better prepare for a visit when cognitive screeners are completed ahead of time. If scores suggest impairment, then the PCC has time to create a plan, whereas if scores are within normal limits, the PCC can focus on other, more important aspects of the visit.

Sabbagh et al [28] reviewed the current landscape of at-home cognitive evaluation tools, barriers to adoption, and associated recommendations for integrating at-home evaluations into clinical practice. The authors argued that, despite current challenges, at-home cognitive testing will likely become an inevitable part of early detection of cognitive impairment, and barriers to the adoption of these tools must be addressed. They noted barriers related to at-home test validation and data quality, user experience (eg, motivation to complete tests, lack of clinical guidance or awareness of at-home screening options, and costs to consumers), and technical approaches (eg, technical fluency of older adults and data security). The authors also noted that patients who are currently experiencing cognitive problems will struggle the most with at-home screening.

At-home cognitive screening (and screenings in general) cannot take the place of a clinical evaluation, particularly when cognitive concerns are present [33]. As such, potential efficiency improvements for clinicians are most applicable for most of their older adult patients who are cognitively healthy and do not require further clinical examination or follow-up beyond an annual screening [3]. Furthermore, some research suggests that healthy older adults prefer to complete cognitive screeners via a computer or smartphone in the privacy of their own home as opposed to paper-and-pencil tests administered by a clinician [16].

### Mobile Apps for Cognitive Screening

Although most at-home cognitive screeners currently available are not yet validated for clinical use, several research tools administered via smartphones show promise for clinical adaptation [28]. The Mobile Toolbox research platform offers a library of cognitive assessments designed for self-administration on a participant’s own smartphone in a completely unproctored setting [34]. Several of the Mobile Toolbox assessments have already demonstrated evidence of feasibility and validity in older adults [35,36], and several more are under validation [37]. Tests in the Mobile Toolbox library measure abilities from a wide range of domains that can be predictive of cognitive health in older adults, including episodic memory, working memory, executive functioning, processing speed, fluid reasoning, and verbal abilities, as well as a variety of patient-reported outcomes [34].

Delivering at-home assessments via smartphones offers several advantages over other devices (ie, tablets and desktops or laptops). Smartphone ownership among older adults has risen from only 10% in 2011 to 61% in 2021, and this trend is expected to continue as the population ages [38]. Moreover, smartphone administration may promote a more equitable reach as low-income earners are more likely to own a smartphone compared with a tablet, laptop, or desktop [39]. Smartphone apps to manage personal health (“mHealth apps”) are becoming increasingly popular, and approximately one-third of older adults use mobile health (mHealth) apps currently [40]. Research also suggests that low-income and minority groups are more likely to access their personal health records exclusively via smartphones [41]. Finally, the mobility of smartphones compared with tablets, laptops, or desktops increases the flexibility of the assessment setting, which may increase accessibility for some patients.

Smartphones offer a unique platform to reach broad and diverse patient populations, but using a smartphone remains a challenge for many older adults. Age-related changes in visual, motor, auditory, or cognitive skills can interfere with older adults’ ability to use smartphone apps effectively [42,43]. Moreover, lower familiarity and comfort with technology may also decrease older adults’ motivation to use these apps [44]. These challenges are compounded when cognitive impairment is suspected [43] and, thus, attention to older adults’ experiences is critical during the design phase of any new technology.

Despite their increase in popularity, most mHealth apps are not optimized for the needs of older adults [45,46]. Research suggests that a collaborative human-centered design process improves the feasibility, acceptability, and usability of mHealth apps for older adults [47,48]. Human-centered design practices focus on the experiences and perspectives of the people whom the technology aims to serve [49]. In the case of health care, multiple stakeholders are often involved in the design process as the success of a new health technology depends on its utility not only for the patient but also for health care providers, caregivers, payers, or other parties. Human-centered design processes are iterative in nature; elements of design are evaluated and refined in frequent successive cycles [50,51]. As such, within this model, the respective design and evaluation processes are intrinsically linked and best examined in tandem.

In this paper, we discuss the iterative, human-centered design of MyCog Mobile, a cognitive screener for remote use by older adults. The goal of designing MyCog Mobile is to leverage the core concepts and cognitive tests (Picture Sequence Memory and Dimensional Change Card Sorting) from the original tablet-based MyCog app while optimizing the user experience and implementation strategy for remote self-administration on a smartphone by older adults. Our design process focused on balancing the needs of older adult patients, clinicians, and clinic administrators. Our research questions for clinicians and administrators were as follows: (1) What would motivate a clinician or clinic administrator to implement a remote cognitive screening process? (2) How might we design a remote cognitive screener to fit well with existing primary care workflows?

Our research questions for older adults were as follows: (1) What would motivate an older adult patient to complete a remote cognitive screener on a smartphone before a primary care visit? (2) How might we optimize the user experience of completing a remote cognitive screener on a smartphone for older adults?

### Study Overview

We iteratively collected and analyzed data from multiple sources (Table 1) between January 2022 and May 2022. First, we spoke with clinicians and clinic administrators to better understand their existing workflows, identify constraints, and envision how a remote screening tool could improve screening practices. We used this information as the foundation for MyCog Mobile’s core features and proposed workflow and to structure our interviews with clinic administrators and patients.

**Figure 1:**
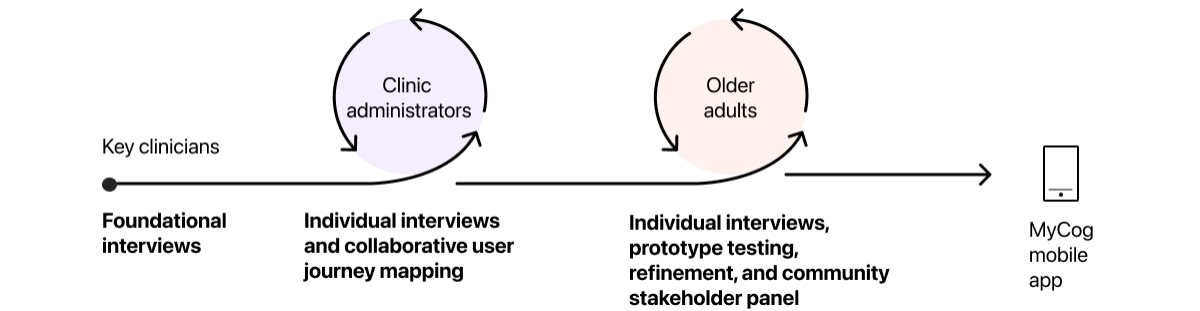
MyCog Mobile design process map.

We then used the human-centered design approach “Design Thinking” to structure our collaborative design process with clinic administrators and older adults [52]. Design Thinking is a cyclical, nonlinear process in which user feedback is iteratively incorporated into the app design (Figure 1). We aimed to better understand and empathize with the user experience through a community stakeholder panel and individual interviews with older adults. On the basis of participant feedback, we developed high-fidelity prototypes of MyCog Mobile, which we iteratively tested and refined to optimize accessibility and usability for the older adult population.

The MyCog Mobile design process was constrained by some predetermined parameters based on the terms of the supporting research grant. First, MyCog Mobile must be designed for remote administration on a personal smartphone. Moreover, the pilot version of MyCog Mobile must be developed for iOS (Apple) devices, with subsequent versions developed for Android and other devices as needed. Finally, the finalized MyCog Mobile versions of the tests must undergo validation in clinical and healthy samples to ensure that the new versions of the tests maintain adequate psychometric properties and diagnostic accuracy.

**Table 1:** Sources of data collected.

Data source	Target demographic	Participants, N	Format
Clinicians	Primary care physicians	5	Formative interviews
Clinic administrators	Administrative staff in primary care settings	3	Semistructured interviews and user journey mapping
Community stakeholders	Adults aged >65 years and caregivers	11	Panel
Individual^a^ volunteers	Adults aged >65 years	5	Individual semistructured interviews, prototype usability testing, and ASQ^b^ and S-SUS^c^

^a^The same 5 individual volunteers participated in semistructured interviews and prototype testing.

^b^ASQ: After-Scenario Questionnaire.

^c^S-SUS: Simplified System Usability Scale.

## Methods

### Ethics Approval

The research procedures were reviewed and approved by the Northwestern University institutional review board (STU00214921). Clinic administrators and older adults who participated in individual interviews provided informed consent verbally and via an electronic REDCap (Research Electronic Data Capture; Vanderbilt University) survey [53,54] and were offered a US $50 Visa gift card as an incentive to participate. Clinician interviews were considered foundational, informal consultations with colleagues and, thus, clinicians were not formally consented or compensated. The community stakeholder panel was facilitated by a Northwestern University center dedicated to community research that was external to the core research team. Thus, panel participants were consented and compensated (with a US $100 Visa gift card) through the external center. We used institutional review board–approved and Health Insurance Portability and Accountability Act–compliant measures to maintain participant confidentiality, privacy, and data security. All data were deidentified, stored on secure servers, and accessed on password-protected devices by trained research team members only.

### Clinician Interviews

We conducted informal, unstructured, 30-minute interviews with 5 clinicians before making any design-related decisions. We recruited clinicians through the authors’ professional networks, and the selection criteria included clinical expertise in geriatrics, cognitive decline, and primary care of older adults. Each clinician was a practicing physician working in different primary care or specialty geriatric settings across the country. The goal of these foundational interviews was to conduct a preliminary evaluation of the feasibility, acceptability, and potential barriers to the adoption of a remote cognitive screener. The discussions focused on themes including current cognitive screening practices and workflows, ideal workflows incorporating a remote cognitive screener, potential benefits and challenges of remote screeners, and technology issues in older patient populations.

### Clinic Administrator Interviews

Next, we conducted semistructured 30-minute interviews with 3 clinic administrative staff members to better understand the practical details of clinical and administrative workflows in primary care settings. These interviews were informed by findings from the informal interviews conducted with clinicians. Clinic administrators were recruited through Northwestern Medicine clinics, and the selection criteria included primary care experience with a large geriatric population and with cognitive screening processes. In total, 67% (2/3) of the participants were practice managers, one in general internal medicine and geriatrics and one in a primary care network with a large geriatric population. The third was a clinical operations manager in the same primary care network as one of the practice managers.

**Figure 2:**
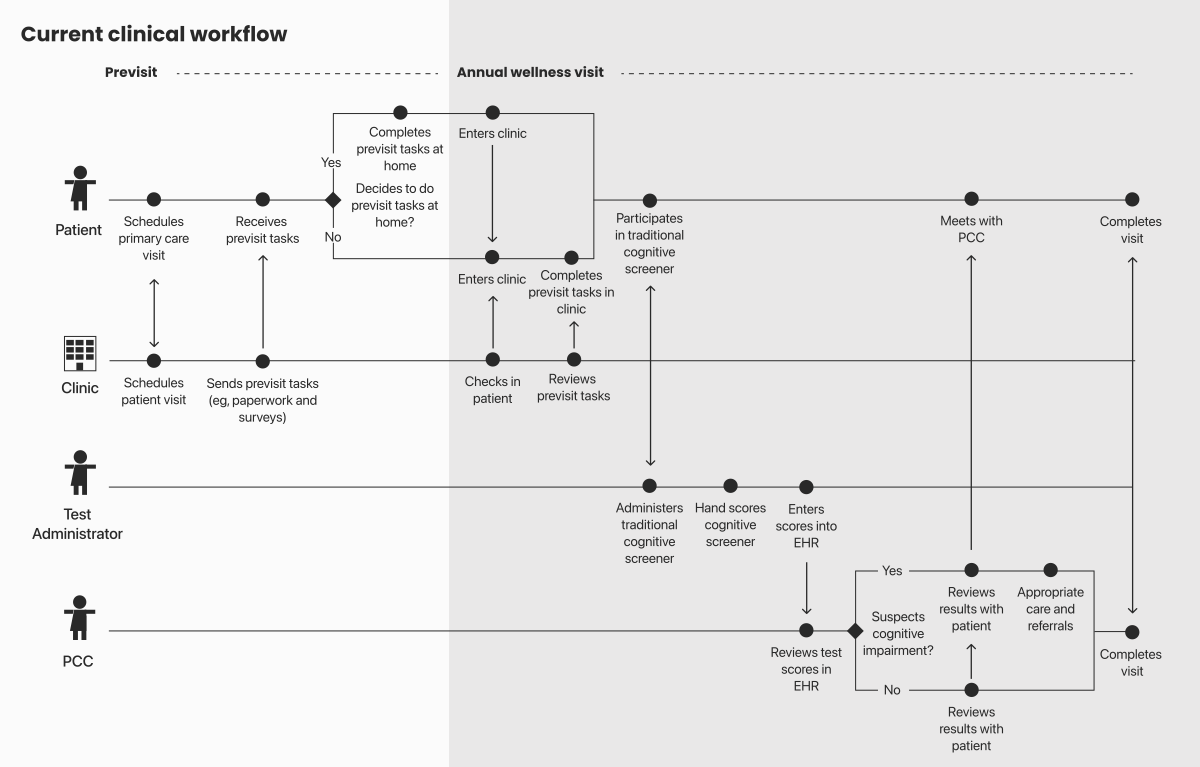
Current multiuser journey map. EHR: electronic health record; PCC: primary care clinician.

**Figure 3:**
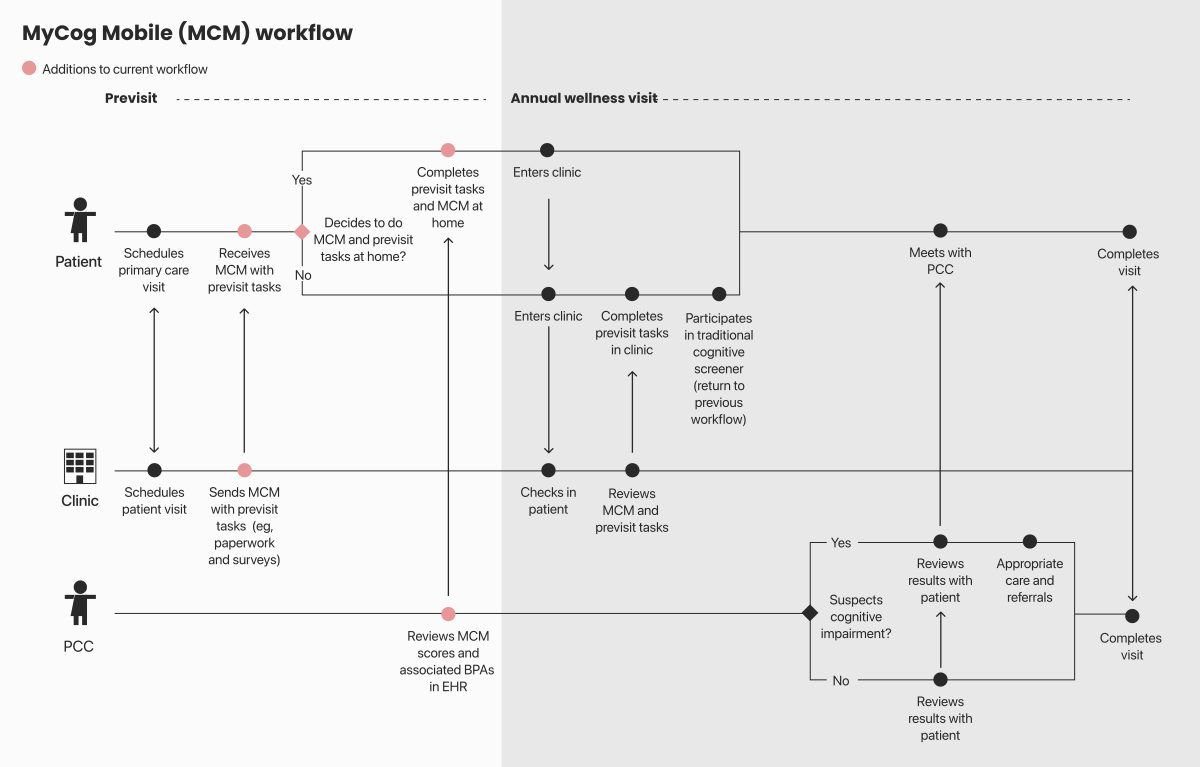
Proposed MyCog Mobile multiuser journey map. BPA: best-practice alert; EHR: electronic health record; MCM: MyCog Mobile; PCC: primary care clinician.

On the basis of administrators’ feedback, we created user journey maps of their current workflow and a proposed workflow including MyCog Mobile. User journey maps are visual models of complex processes and interactions that can be used to represent health care services from the perspective of different stakeholders [55]. We focused on the broad tasks and interactions necessary for multiple users (patients, support staff, and clinicians) to complete a cognitive screening in a primary care setting. Visual models of the user journey maps (Figures 2 and 3) were shared with the administrators following the interviews for further feedback, refinement, and confirmation of the final models.

### Older Adult Interviews

Older adults were recruited from the research participant recruiting platform User Interviews (User Interviews Inc). Eligibility criteria were age of ≥65 years, ownership of an iOS smartphone, and access to the videoconferencing app FaceTime (Apple Inc; see Table 2 for participant demographic information). The requirement for iOS smartphone ownership was based on a grant constraint that the original version of MyCog Mobile be built for iOS mobile phones if a native app solution was pursued. Participants were not asked to report their cognitive health status, although all participants were cognitively able to complete the session tasks.

**Table 2:** Individual interview and prototype testing participants.

ID	Age (years)	Gender	Prototype version number	Race or ethnicity	Education	Employment
P1	74	Woman	0	White	Master’s degree	Retired
P2	78	Woman	0	White	Master’s degree	Employed part-time
P3	65	Man	1	Mixed-race Hispanic or Latinx and Asian	Bachelor’s degree	Employed part-time
P4	70	Woman	2	White	Some college	Retired
P5	69	Woman	3	Black or African American	Bachelor’s degree	Retired

Semistructured interviews were conducted over FaceTime and recorded with the participants’ consent. The first part of the interview consisted of discussions of the participants’ experiences, attitudes, preferences, and beliefs about cognitive screening and the possibility of completing a screener at home on their smartphone. In the second part of the interview, participants interacted with and provided feedback on the prototype at various stages of design (as described in the *Prototype Testing* section).

The interviews were transcribed using the automated transcription software Otter.ai [56] and anonymized. The first author (SRY) conducted an inductive, reflexive thematic analysis of the transcripts, including familiarization with the data, generating codes relevant to study aims, and then grouping codes and developing broader themes [57]. We used functions in Otter.ai [56] and Trello (Atlassian) [58] software for coding and thematic grouping.

### Stakeholder-Academic Resource Panel

Stakeholder-Academic Resource Panels (ShARPs) are custom panels run by a university-affiliated organization that bring together community stakeholders with personal experience related to research projects. These community members offer feedback on adaptations that can improve research relevance, feasibility, and dissemination opportunities. The goal is to engage communities in research and foster inclusive design of research studies and innovations. ShARPs consist of 90-minute sessions conducted over Zoom by a professional moderator external to the core research team.

The ShARP included 10 adults aged >65 years and 1 caregiver, all of whom resided in the Chicago metropolitan area where MyCog Mobile will be piloted. The moderator recruited panel members through fliers that provided a brief description of the research project as well as direct outreach to an established network of community members. In total, 2 members of the core research team (SRY and CN) were present to explain the study background and observe the discussion. The moderator facilitated a discussion of the benefits and challenges of cognitive screening on a smartphone for older adults. Participants also watched short videos of examples of downloading a native app or accessing a screener via a website, as well as a video of the last version of the MyCog Mobile prototype (version 4). Participants were able to use a polling tool during the session to provide feedback on their preferences regarding how to access the screener (ie, native app, website, or no preference). The moderator recorded the session and provided the research team with structured notes. Although one of the goals of the ShARP program is to recruit diverse groups of participants that are representative of the larger community, they did not disclose specific demographic information about the participants beyond the general age range (≥65 years).

### Prototype Testing

We used an iterative prototyping process to test and refine the designs based on user feedback. The Picture Sequence Memory and Dimensional Change Card Sorting tests from the National Institutes of Health Toolbox Cognition Battery [34] were used as the “version 0” prototype as these tests would serve as the foundation for the MyCog Mobile screener. As such, version 0 prototype testing was conducted using the live Mobile Toolbox app. All other versions were clickable prototype mock-ups made using Figma design software (Figma, Inc) [59]. All usability testing was conducted on participants’ personal iOS devices.

During individual interviews with older adults, the researcher sent participants a link to the prototype via SMS text message and posed a realistic scenario in which the participants’ primary care clinic asked them to complete the screener before an appointment. The participants either downloaded the live Mobile Toolbox app (version 0) or followed the link to the Figma design prototype (versions 1-4). They then shared their screen via FaceTime so the researcher could observe their interactions and solicit feedback. Following the interview, participants completed usability measures via REDCap [53,54], including the Simplified System Usability Scale (S-SUS) [60] and After-Scenario Questionnaire (ASQ) [61].

The S-SUS is a modified version of the original System Usability Scale designed for adults aged >65 years with or without cognitive impairments [60]. Participants rate their level of agreement with statements about their experience using a given system (in this case, a prototype of MyCog Mobile) on a 5-point Likert scale. Several independent studies on the original System Usability Scale have demonstrated evidence of its internal consistency, sensitivity to change, and concurrent validity with other usability measures [62]. A total score of ≥80 (out of 100 possible points) is considered above-average usability [62].

The ASQ is a 3-item questionnaire that prompts participants to rate their satisfaction on a 7-point Likert scale after using a system in a hypothetical scenario that asks them to complete realistic tasks [61]. In the case of MyCog Mobile, patient participants were prompted with the following scenario before using the prototype: “Imagine that you have an upcoming appointment with your doctor for an annual checkup. The clinic has asked you to complete some cognitive screening activities on your smartphone before your appointment and sends you this link.” The ASQ has demonstrated evidence of internal consistency; concurrent validity; and sensitivity to different systems, user groups, and scenarios [61]. Total scores range from 1 to 7, with higher scores indicating higher levels of satisfaction.

## Results

### Clinician Interviews

The clinicians we spoke with were motivated to introduce a new cognitive screening paradigm if the process could save them time while maintaining diagnostic accuracy. These discussions revealed several important preferences and constraints that informed the MyCog Mobile design process and proposed implementation, including the following: (1) clinicians felt strongly that proctored remote screening would be impractical in existing clinical workflows given the constraints on time and resources within primary care settings; (2) clinicians require a screening test with strong evidence of validity in remote self-administration contexts; (3) clinicians want to see results that automatically populate the electronic health record (EHR) as opposed to manual score entry that would require additional staff or clinician effort; (4) clinicians want to see simplified, easily interpretable score reports tied to clinical recommendations; and (5) clinicians had concerns about their older patients’ ability to navigate a smartphone-enabled cognitive screener on their own.

### Clinic Administrator Interviews

Similar to clinicians, clinic administrators were interested in a remote cognitive screener that could help save them time and reduce their workload. They noted that staff are already responsible for conducting cognitive screenings and would welcome changes in cognitive screening processes that promote greater efficiency. Importantly, 67% (2/3) of the administrators noted that their current screening process only takes approximately 5 to 10 minutes to complete. Thus, a new screening process must involve very little time and effort on the part of the staff to be compelling.

We used the existing workflows described by administrators to model a user journey map (Figure 2). Although administrators worked in different roles and settings, their cognitive screening workflows shared features that are captured in the user journey maps. In these settings, support staff typically conduct cognitive screeners using paper-and-pencil measures (ie, Mini-Cog [63] or Montreal Cognitive Assessment [MoCA] [64]). Staff then hand score these protocols and manually enter scores into the EHR for the PCC to review. Administrators noted that, in their current workflows, previsit tasks are typically sent electronically to patients before their appointment, such as an e–check-in, Medicare wellness questionnaire, or consent forms. They also noted that some patients do not complete the tasks before the visit and may complete these tasks after the physical check-in at the clinic.

### Older Adult Interviews

We constructed six themes based on the feedback from individual interviews with older adults: (1) annual cognitive screening provides reassurance, (2) older adults expect additional benefits to using a smartphone-based screener before a visit, (3) older adults want to be informed by their clinic about the purpose and process of cognitive screening, (4) older adults prioritize security and simplicity in mHealth apps, (5) older adults have concerns about using smartphones for screening because of the screen size, and (6) older adults tend to defer to their PCCs’ requests.

#### Theme 1: Annual Cognitive Screening Provides Reassurance

Although only 20% (1/5) of the participants had been offered the opportunity to complete a cognitive screening in the past, 80% (4/5) of the older adult participants were familiar with the concept of cognitive screening. All participants reported that they would complete a cognitive screener at home on a smartphone if their PCC asked them to. Participants were motivated to complete cognitive screeners (both in person and remotely) to learn about their cognitive health status. For example, when asked why he would participate in a cognitive screening, participant 3 responded, “I’d like to know, you know, if I’m losing my marbles.” Participants felt that cognitive screeners could provide reassurance that they were cognitively healthy even when they were not concerned about their cognitive functioning. Participant 1 said that she would complete a screener “because I want to make sure that my brain was functioning properly even though in my mind, I know it is. But don’t we all think that even when it’s not?” Participants also recognized some benefits of early detection if a screening result was positive; for example, participant 5 said that “I would do [a cognitive screener] so I can get some kind of assistance if needed or intervention.”

#### Theme 2: Older Adults Expect Additional Benefits to Using a Smartphone-Based Screener Before a Visit

Beyond the benefits of cognitive screening generally, participants reported that at-home screeners specifically could help save them time during their in-person visit and feel more private. Participant 1 said that “I’d like to do [a cognitive screener] at my own convenience and in a comfortable place.” Participant 5 said that she would like the opportunity to think about the idea of completing a cognitive screener at home rather than being surprised by it during her clinic visit. She said that “It would be nice to have a heads up before you meet with the doctor, so you can kind of wrap your head and mind around it.”

Some participants expected to see their results within the app immediately after completing the screener and disliked the idea of waiting until their appointment to learn their results. Participant 3 said the following:

I think I’d probably like to know sooner [than my appointment] because then you’re worrying. Not worrying, but you know, you’re curious and you may not see the doc for a couple of weeks.

Participant 5 felt that the effort required to complete the screener before the visit warranted instant feedback, saying that “if I take the time to do it, I want immediate results. I don’t want to have to wait.” However, all of these participants said that they would still complete the screener if they knew they had to wait for results and understood why waiting might be necessary. Participant 1 said the following:

It wouldn’t make sense if someone was struggling with [the screener] to give them very bad results without someone there to say, “Let’s talk about this.”

#### Theme 3: Older Adults Want to Be Informed by Their Clinic About the Purpose and Process of Cognitive Screening

Some participants reported that they would be concerned if a clinician asked them to complete a cognitive screener before a visit; they worried that the request may indicate that the PCC suspected they already had a cognitive problem. Speaking of the importance of providing the rationale for screening, these participants reported that they would feel better if they knew that the test was routinely administered to all patients aged >65 years, including healthy people. For example, participant 2 said the following:

Yeah, if it was part of your annual exam, then I would [complete the screener], but it would kind of make me nervous, knowing that [my doctor] wanted me to do it without knowing why.

#### Theme 4: Older Adults Prioritize Security and Simplicity in mHealth Apps

Participants in our sample were familiar with using mobile apps and websites through their smartphones. They were also familiar with communicating with their clinics (via a patient portal or email) and completing previsit tasks electronically. Participants expected that an at-home screener would be completed electronically and their clinic would send them a secure link to access the screener via email or their patient portal. They indicated that they would want to be sure that the link was sent by their clinic before accessing the screener. Participant 1 said that “I would probably trust [a link to an app] if it just came from my physician.” In total, 40% (2/5) of the participants noted that it would be easiest for them to receive the link to the screener through their patient portal apps as they already frequently used these apps to manage appointments and communicate with their physicians.

Participants in our sample preferred a remote cognitive screener that required only a few steps to access and use. For example, participant 4 said that she would want to access the app in as few clicks as possible, stating that “You could produce the link to download, you know, press the button, download it. Boom! There it is.” Difficulty with passwords was also noted to be a potential source of complexity and frustration. Participant 1 said the following:

You want it to work, and you want it to be simple, and not something that frustrates you because it throws you out or because it didn’t like the way you logged in.

She explained that she felt frustrated when she had to create complicated passwords with many requirements (eg, combinations of capital and lowercase letters, numbers, and symbols).

Participants did not have a strong preference between downloading a native app to their phone or accessing the screener through a web application and were familiar with both processes. Some participants felt that downloading native apps cluttered their phones but also expected to delete the app from their device immediately after completing the screener. Participants reported that an ideal app would need to be functional and free of bugs. Participant 2 said that she would look at reviews of a native app before downloading it to learn “what people thought about it, you know, like if it is actually bogged down, if it was slow, if it actually worked.” Of note, participant 1 pointed out that the device type played an important role in her comfort with downloading a native app, saying that “I’m not real hesitant to download apps on an Apple device because to me, they seem very, very safe as far as viruses and all that go. If I had a galaxy, I might think twice about it.”

#### Theme 5: Older Adults Have Concerns About Using Smartphones for Screening Because of the Screen Size

Most participants had concerns about being able to navigate text and images on a small smartphone screen and suggested that a tablet or computer would be preferred. Participants were willing to accept a smartphone app if these concerns were addressed in the user interface design. For example, participant 1 said the following:

I would want it on an app with reasonably sized text and easy-to-push buttons. If it’s easy to click buttons, that’s fine, it doesn’t matter

if the screener is on a smartphone

When asked what would make completing the screener on a smartphone easiest, participant 5 responded, “I guess typically, it would be as little keyboarding as possible.” She explained that she did not mind typing on a computer keyboard, but typing on a smartphone keyboard was more tedious.

#### Theme 6: Older Adults Tend to Defer to Their PCCs’ Requests

Despite the aforementioned concerns, participants in our sample were willing to complete nonpreferred aspects of the screening process to comply with their PCCs’ requests. Participants trusted that their PCCs ordered the screener with optimal patient care in mind. For example, when asked if she would complete the screener before a visit despite her concerns about why her PCC had ordered it, participant 2 responded, “Probably, I mean, if my doctor asked me to do it, then yes.” She explained that she would feel more confident about completing the screener knowing it was a routine best practice and she could discuss the feedback with her physician. Participant 5 said that she would prefer to see immediate results within the app but would still complete the screener if she had to wait. She explained that she would complete it anyway “because I think it’s an assessment that is necessary as we age, you know, like other health things.”

### ShARP Interviews

The ShARP session moderator posed a set of questions to the group to guide discussion on cognitive screenings and the proposed MyCog Mobile project. Much of the feedback provided by the panel participants was consistent with that provided by the individual interview participants. When asked about their familiarity with cognitive screenings, many panel participants were unfamiliar with the practice of routine screenings in primary care. Patients were unsure whether their PCCs were conducting cognitive screenings, and some expressed concerns that their PCCs had not mentioned routine screenings during their annual visits.

When asked about the potential benefits of completing a screening on a smartphone at home, a participant shared that they would feel more comfortable completing the screening in the privacy of their own home compared with in a clinic. Another participant felt that completing the screening at home would free up time to discuss other health issues with their physician during their visit. Finally, participants agreed that completing a cognitive screening in general provided reassurance that they were cognitively healthy.

Participants also identified potential disadvantages of completing a remote cognitive screening on a smartphone. All participants (11/11, 100%) reported that they owned and were comfortable using smartphones and apps but knew others their age for whom that was not the case and, thus, who would not be able to participate in the remote screening process. Participants expressed concerns about the security of their health information being transmitted over a smartphone and were wary of scams aimed at older populations. A participant said that she believed that some older adults may avoid taking a screening test for fear of learning that they may have a cognitive problem and risk losing their independence.

Participants offered ideas on how to make a smartphone screening process easy. Similar to the individual interview participants, panel participants also noted that they often forgot passwords and simplifying the password process would be helpful. They suggested that it would be helpful to allow caregivers to help patients access the screener, such as helping with downloading an app or resetting a password. A participant indicated that she would prefer to complete the screener on a laptop instead of a phone as a bigger screen is easier to navigate. Participants noted that it would also be helpful if their clinic offered some sort of orientation or training on how to complete the screener.

Participants in our community research panel sample were familiar with the process of downloading an app. Participants were polled about whether they would rather access the screener by downloading a native app to their smartphone or visiting a website (web application) in their browser. In total, 73% (8/11) of the participants voted that they would prefer to download a native app, whereas 18% (2/11) voted for a website, and 9% (1/11) had no preference. Most participants felt that an app would be the most secure way to complete the screener, and a participant noted that the iOS App Store offers assurance that the available apps are safe.

Participants agreed that the most important access issue for them was knowing that the screening was sent from a trusted source (their PCC). A participant shared positive experiences in using their PCC’s patient portal app because of its perceived security and trustworthiness. Participants also indicated that factors such as access to wireless internet and available storage space on their smartphones would influence their preferences for a website or native app.

### Prototype Testing

**Figure 4:**
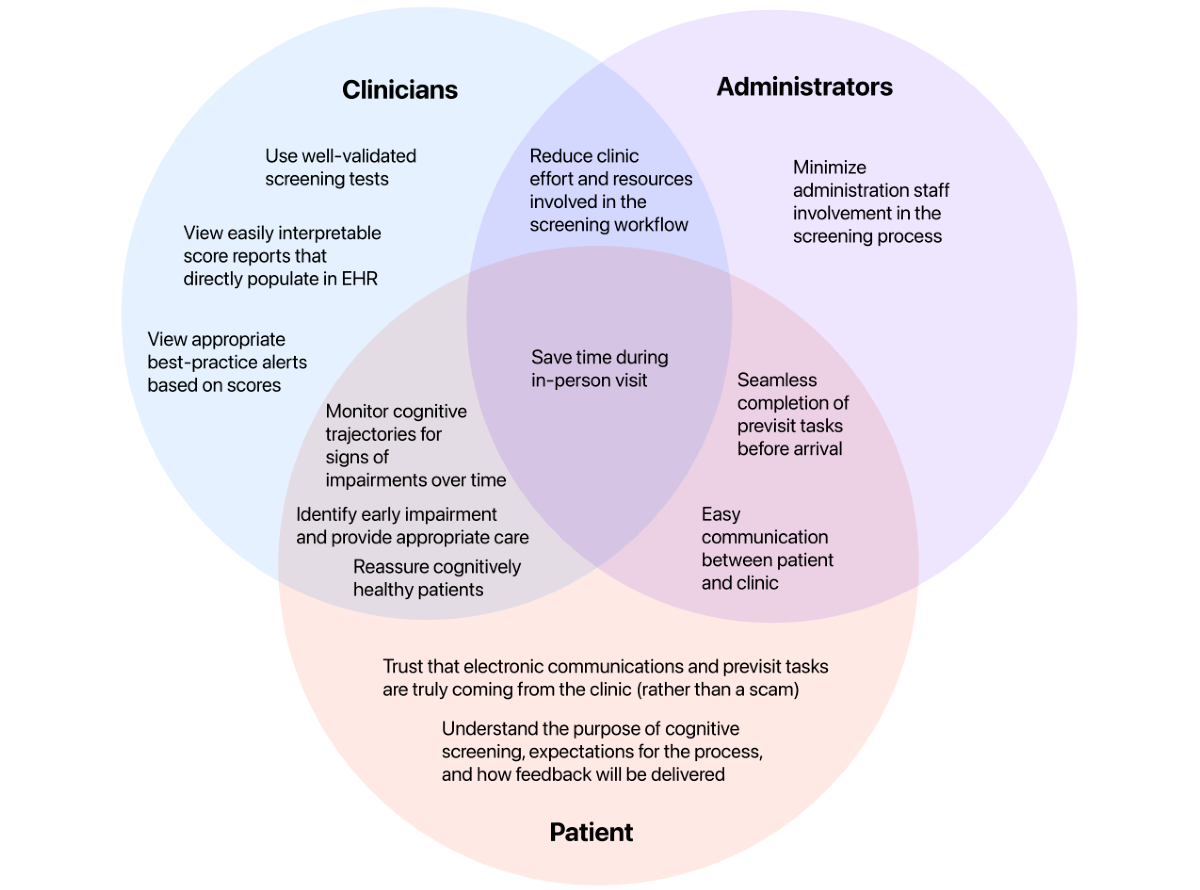
Relevant stakeholder needs in a primary care cognitive screening process. EHR: electronic health record.

A total of 4 versions of the MyCog Mobile prototype were created and tested, with most improvements made in the transition from version 0 (the Mobile Toolbox versions of the tests) to version 1 (the first Figma prototype of MyCog Mobile). The usability issues noted by participants were addressed in each subsequent version of the prototype (Table 3). We defined usability issues as any difficulty that a participant encountered in the prototype that would negatively affect their ability to complete MyCog Mobile in a real-world context or their satisfaction with the user experience [65]. When applicable we also considered the overlapping and diverging needs of clinicians, clinic administrators, and older adults when incorporating usability feedback (Figure 4). The mean score on the S-SUS was 91 (SD 5.18; range 85-97.5), indicating above-average usability for each version of the prototype. Similarly, the ASQ average score was 6.13 (SD 8.40; range 5.33-7.0), which indicates an above-average level of satisfaction with using the prototypes in the imagined scenario. Screen recordings of the introduction and practice items for the tests are included in Multimedia Appendices 1 to 4.

**Table 3:** Usability issues and prototype revisions based on usability testing.

Version number	Changes from previous version	New usability issues identified
0	N/A^a^	Animation demonstrating the task was confusing, and participants expected to interact with the animation Transition from 4-picture practice item to 14-picture live items on PSM^b^ felt drastic Navigating to the next activity from the menu was confusing and added unnecessary clicks to the workflow Landscape view made it easy to accidentally swipe out of PSM Text was too small Navigation arrows to move forward were ambiguous Delay between the end of audio instructions and perceived start of the activity causes false starts in PSM
1	Demo animation removed and instruction and practice rounds merged to provide an interactive learning experience in both tasks 8-picture practice item included to gradually prepare participants for the live item in PSM Removed navigation menu so that the first activity flows seamlessly into the next PSM redesigned for portrait view Text size increased End of audio instructions aligned with start of PSM live items Welcome screen added to explain the purpose of screening, provide instructions, and set expectations for results (Figure 5) Exit screen added with instructions to delete the app and reminder that the results will be discussed during the visit (Figure 6)	Participants noted difficulty with dragging pictures in PSM Potential frustration with being forced to “Try Again” on the PSM 8-picture practice item
2	Option to drag or tap to arrange pictures in PSM “Try Again” or “Move On” options added after errors on practice items	“Try Again” button obscured error feedback in PSM practice item Tapping preferred to dragging in PSM Participant unaware that tapping was an option
3	PSM instructions updated to make tapping gestures explicit (Figure 7) “Try Again” button floats in from the top of the screen, so error feedback is visible (Figure 8) Dragging functionality removed	No significant usability issues identified by participants
4	Education question added to improve predictive validity Navigation buttons labeled with words instead of symbols (“Next,” “Back,” and “Help”)	No significant usability issues identified by participants

^a^N/A: not applicable.

^b^PSM: Picture Sequence Memory.

**Figure 5:**
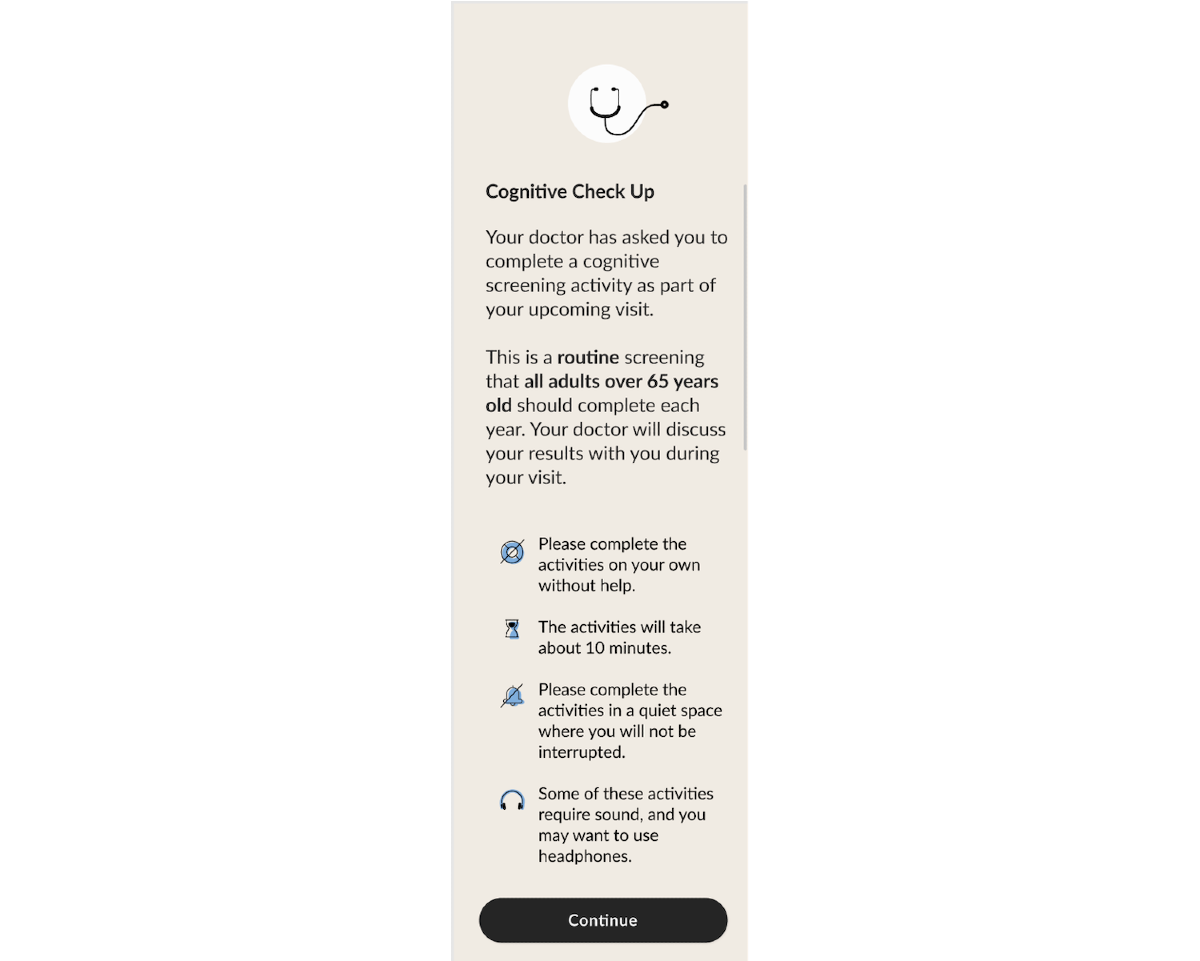
Introduction screen.

**Figure 6:**
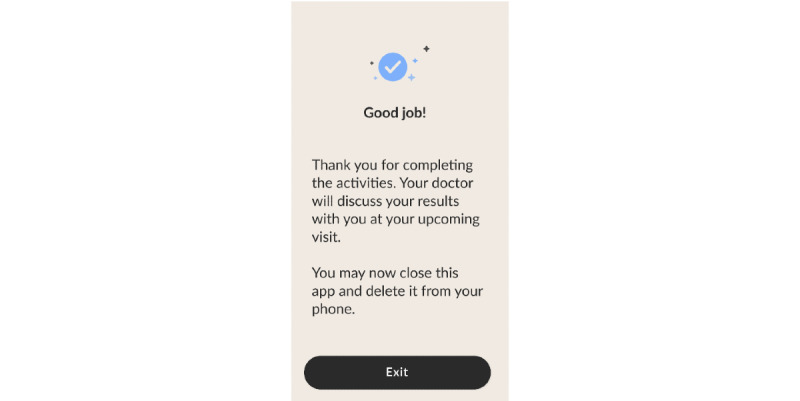
Exit screen.

**Figure 7:**
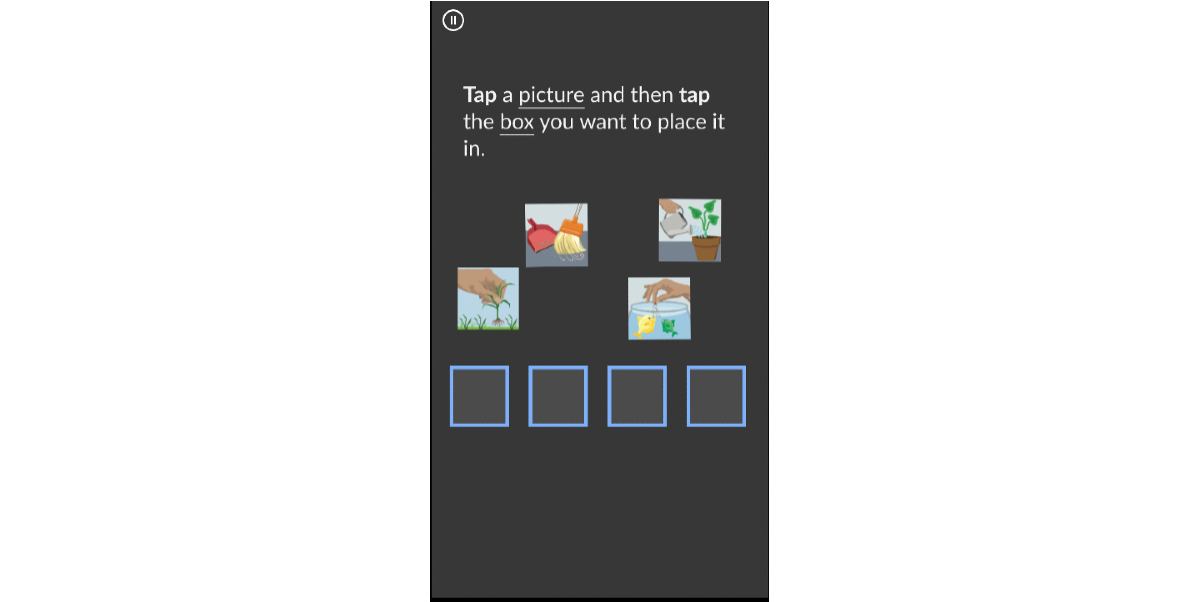
Picture Sequence Memory tapping instructions.

**Figure 8:**
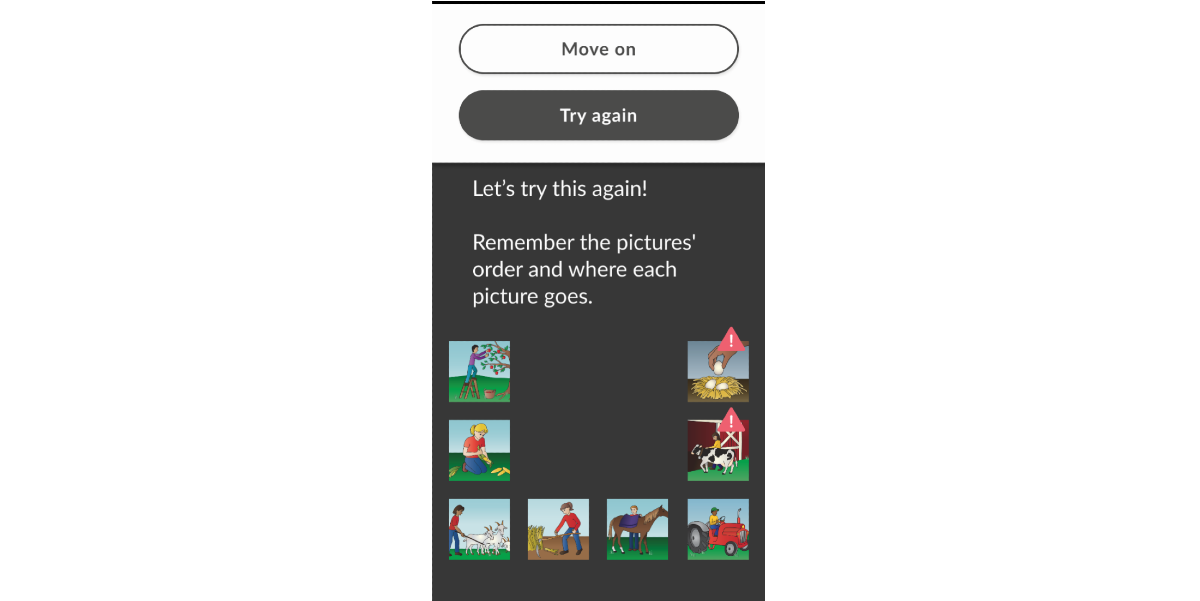
Picture Sequence Memory practice item feedback.

## Discussion

### Principal Findings and Design Implications

#### Overview

Through an iterative, human-centered design process, we developed a viable remote cognitive screening app and proposed an implementation strategy for primary care settings that was optimized for multiple stakeholders. On the basis of individual interviews and collaborative user journey mapping, we found that clinicians and clinic administrators were motivated to use a remote cognitive screener if it could save them time. Minimizing staff involvement and maximizing EHR integration were key implementation factors to ensure that MyCog Mobile will fit in well with existing primary care workflows. Through individual interviews and a community stakeholder panel, we found that older adults were motivated to complete a remote cognitive screener to ensure that they were cognitively healthy or receive intervention if needed. They perceived additional benefits to at-home smartphone-based screening but also reported some concerns that required mitigation in the app design. We iteratively designed and tested high-fidelity prototypes that incorporated a series of usability improvements to optimize older adults’ user experience, primarily aimed at simplicity of instructions and app workflow and transparency regarding expectations of the screening process.

#### Clinician and Clinic Administrator Perspectives

Clinicians and clinic administrators perceived that the primary benefit of MyCog Mobile was reducing in-clinic time and effort spent on screening healthy older adults, who comprise most of their patients. They reported that a portion of their patients can use smartphones and complete previsit tasks electronically through patient portals, which was consistent with the experience of older adult smartphone users in our sample. Although current screening processes are relatively brief, they require substantial staff involvement and several steps that introduce opportunities for error (eg, manual scoring and data entry). Beyond time saved directly by removing the need for in-person staff administration, automation of the MyCog Mobile scoring and reporting processes can save additional time when implemented on a large scale.

Despite the overall efficiency improvements, clinicians and administrators also recognized that patients at the highest risk of dementia (eg, older age [66] and less education [67]) may have the most difficulty using MyCog Mobile, which is an important limitation of a remote screening paradigm. Regardless of screening results, older adults with cognitive impairments will need, at a minimum, a follow-up in-person evaluation to confirm a diagnosis and receive referrals and appropriate care. As such, the efficiency gains related to remote screening practices are most relevant to patients who are healthy. Starting a routine remote screening process while patients are still healthy allows clinicians to see not only changes in scores over time but also changes in a patient’s ability to complete the screener at home.

It is also likely that some older adult patients will not complete a screener for reasons unrelated to cognitive impairment. Administrators in our sample reported that a portion of patients who are healthy do not complete previsit tasks at home currently. Understanding why a patient did not complete the screener remotely may be a source of clinical information. For example, did the patient struggle with the smartphone screener because of a lack of technological experience or because of a cognitive problem? For patients who do not complete MyCog Mobile at home, clinics will need to return to their existing in-person screening process, as shown in Figure 3. Although MyCog Mobile can be used alongside any in-person screener, clinics that adopt MyCog Mobile may also consider using the MyCog tablet app for in-person screenings to maintain relative consistency in test selection across patients.

Interviews with clinical stakeholders helped us identify crucial constraints and features necessary for MyCog Mobile to improve workflow efficiency. Critically, we learned that clinicians viewed proctoring a remote screening as prohibitively time-consuming in the context of their current workflows even if conducted by a medical assistant or other support staff. On the basis of this finding, our team chose not to pursue the design and development of costly remote proctoring features such as bidirectional video communication, which would not be viable in practice.

Both clinicians and administrators stressed the importance of integrating every aspect of the cognitive screener into the EHR to improve efficiency. On the basis of administrator feedback, we created a proposed MyCog Mobile user journey map (Figure 3) intended to leverage existing clinic-patient interactions and minimize additional tasks for both patients and staff. Ideally, a link to the screener would be sent to a patient automatically along with the other previsit tasks they are already asked to complete electronically (eg, e–check-in, insurance verification, and consent). Patients would then accept the MyCog Mobile screener as part of the tasks they need to complete before their appointment. At the time of the visit, the support staff would only need to check whether the screener was completed and would no longer need to be involved in the screening process. A completed screener upon arrival not only frees time for busy staff but also minimizes the possibility for errors in manual scoring or data entry when scores are automatically calculated and transferred to the EHR [23,24].

Integration with an EHR addresses clinician-specific needs as well. First, automatically populating scores into the EHR eliminates the need for clinicians to find and review a separate score report (eg, a paper protocol or separate software application), which would add additional steps and time to their workflows. Configuring automated best-practice alerts within the EHR may help reduce the cognitive load of clinical decision-making for PCCs following a positive result [5]. Clinicians also noted that it would be helpful to review screener results in the EHR well before a patient arrives so that they have additional time to plan for the visit, especially if impairment or decline is suspected.

#### Older Adult Perspectives

Individual interviews and the community research panel session revealed that older adults are motivated to complete cognitive screeners to ensure that they are cognitively healthy or receive early care if they need it. Participants perceived additional benefits of screening at home, including saving time during their in-person visits and privacy. At the same time, some participants worried about why their PCCs would ask them to complete a screener or what poor performance might indicate. To feel comfortable completing MyCog Mobile, participants first wanted to understand why their PCC had requested them to do so and when they could expect feedback. They also wanted to be sure that the request was sent from their primary care provider and not from an unsafe source. They worried that completing a screener would be difficult on a small smartphone screen and suggested some related design improvements. Despite some concerns, the older adults we spoke with were willing to comply with nonpreferred aspects of the remote smartphone screening process as they trusted that their PCCs ordered it with their best intentions in mind.

The ways in which patient needs and goals for a cognitive screening process in primary care overlap with and diverge from those of clinicians and administrators are important to consider (Figure 4). Patients, clinicians, and administrators share a common goal of efficiency in in-person primary care visits but may be motivated to achieve this goal for different reasons. Both clinicians and patients want to prioritize spending visit time on the issues that have the most impact on health and quality of life [68] and simultaneously want to ensure that they are not missing a nonobvious health problem that could be caught by a routine screening [69-71]. However, clinicians’ need for efficiency is also driven by the need to accommodate large daily patient loads, which can limit the time spent on individual patients [72]. Similarly, clinic administrators have many tasks to complete related to each patient visit and need to ensure that visits are completed in an efficient and timely manner [73]. As such, clinicians’ and clinic administrators’ goals for efficiency may not completely align with those of a patient. For example, a patient may prefer to complete a screening in person or not participate in a cognitive screening at all [71].

Although we found that older adults in our sample were willing to defer to the requests of their PCCs even if aspects of the screening process did not align with their preferences, clinics should be careful not to take advantage of patients’ trust. There are times when clinics must sacrifice efficiency to accommodate patient needs and provide optimal patient-centered care. Ultimately, individual clinics need to decide how to implement MyCog Mobile, communicate the process to their patients, and support app use based on the needs of their unique patient populations. We intend to collect data on how clinics can best implement MyCog Mobile to maximize compliance, patient experience, and the potential benefits of MyCog Mobile during an upcoming validation and feasibility study.

#### MyCog Mobile User Experience Design

Concerns expressed by older adults in the individual interviews and ShARP directly informed our user experience design. First, we designed introduction and exit screens to set expectations for the remote screening process, address common concerns, and provide clear and simple instructions (Figures 5 and 6). The introduction screen emphasized the routine nature of the screening and notified the user that they would learn about their results at their upcoming visit. Instructions were written for a middle school reading level or lower and assessed for ease of potential translation in subsequent versions (eg, simple, direct statements free of idiomatic or culture-specific language). Users are instructed to complete the screener in a distraction-free environment and given the option to delete the app upon completion. We also increased the size of the text and images and did not include typing to optimize the app for older adults’ preferences on a mobile screen.

To address older adults’ concerns regarding the screener being sent from a legitimate source, we proposed that clinics send a link to the screener to the patient via an established communication channel. An EHR-based patient portal message is an ideal route for many clinics if the patient has a patient portal account (eg, Epic’s MyChart); however, a link could be sent via a secure email or SMS text message if that is how the clinic usually communicates with patients. Clinics will need to carefully consider how they communicate with their patients about the MyCog Mobile process when sending the link to the app to ensure that patients trust the source and understand the process.

Participants generally perceived native iOS apps as more secure and trustworthy than web applications and were familiar and comfortable with downloading apps. To meet the needs of both clinical and patient stakeholders, we proposed a native app solution that could be integrated with a patient portal. A link to download the app could be sent via a patient portal message similar to familiar previsit tasks such as e–check-in. The perceived and actual security of non-iOS apps should be considered if versions of MyCog Mobile for other devices are pursued in the future.

Creating and remembering passwords was a source of frustration for many patients. We proposed an open authorization process so that patients could use their patient portal credentials to log in to MyCog Mobile, which not only simplifies the log-in process for the patient but also serves the clinician’s goal of automatically transferring score data back to the EHR. It is likely that many patients will need to find or reset their patient portal passwords or create new accounts, which may present barriers to using the app. However, the extra steps to find or create new patient portal credentials are likely similar to what would be necessary to create new MyCog Mobile credentials but offer the benefit of broader patient portal access and use.

Findings from iterative prototype testing identified several usability issues that led to incremental improvements in the MyCog Mobile user experience design. Overall, high levels of satisfaction with each version of the prototype during usability testing and in follow-up surveys suggested good usability of the MyCog Mobile user interface even at baseline (the Mobile Toolbox versions of the tests). The most usability improvements were made in the transition from the Mobile Toolbox version (version 0) to the first version of the MyCog Mobile prototype (version 1). Many of these changes were consistent with best practices for mobile app design for older adults, including increasing text size; gradually increasing difficulty level in the practice rounds; and creating simple, interactive instructions [46].

We also removed the activity menu in favor of a seamless flow through the activities to encourage patients to complete both tasks in 1 session and reduce the number of clicks necessary to complete the screener. In later versions of the prototype, we learned that dragging pictures to arrange them in the Picture Sequence Memory task was difficult for some users and could be problematic for older adults [74,75]. Thus, we changed the touch screen interaction gesture to tapping the images instead of dragging them to improve accessibility (Figure 6). After completing the prototype testing, all participants reported that they felt confident in their ability to complete the MyCog Mobile screener on their own before a primary care visit.

### Comparison With Prior Work

To our knowledge, this is the first detailed description of an iterative design and evaluation process for an unproctored cognitive screening app for primary care settings, and we hope that this work can serve as a model for researchers and mHealth app developers interested in designing similar tools. Our findings were consistent with research regarding older adults’ perceptions of and willingness to participate in cognitive screenings [16,17,71]. Moreover, the MyCog Mobile paradigm aligns with previous research regarding healthy older adults’ preferences for completing cognitive screeners on electronic devices in their own homes [16].

Our proposed implementation strategy and clinical workflow may help overcome some of the barriers to remote screening for healthy older adults noted by Sabbagh et al [28]. Presenting MyCog Mobile as a previsit task to be completed by patients before a primary care visit provides patients with access to home-based cognitive screeners that they otherwise may not have known existed. Crucially, the MyCog Mobile screening process ensures that patients will receive feedback and guidance about the results of their screener from a qualified clinician during their in-person visit. This process reduces the potential harm that may result from unsupervised screening and immediate score reporting that could occur with mHealth apps. Using MyCog Mobile in the context of a Medicare annual wellness visit will also address the authors’ concern about the costs and reimbursements associated with cognitive screening as annual cognitive screening is covered by Medicare and most other insurance providers.

Findings from our prototype testing and subsequent design improvements were largely consistent with the suggested best practices by Wildenbos et al [42,43] for mHealth app usability for healthy older adults, including addressing the cognitive, physical ability, perceptual, and motivational barriers that can prevent older adults from using apps. Although most mHealth apps aim to be as easy to use as possible, MyCog Mobile is unique in that its core function is to test cognitive abilities and, thus, the difficulty of the activities themselves is fixed. However, we aimed to minimize the cognitive load of the instructions and navigation of the app by breaking down the instructions into simple, interactive steps and creating a seamless flow through the activities [46]. We also addressed perceptual and physical barriers by increasing text and button size, replacing the dragging gesture with tapping, and indicating the use of headphones for activities with audio.

Wildenbos et al [42,43] noted that, among the barriers to mHealth app use for older adults, motivational barriers are the least often addressed in usability design. Our study is relatively unique in that questions about older adults’ motivation were central to the design and evaluation process. Our proposed design aims to leverage the potential benefits of remote cognitive screening from the older adult patient’s perspective, and we encourage clinics to consider older adult motivations as well when introducing MyCog Mobile to their patients.

It is important to note that an in-depth qualitative study of attitudes toward the cognitive screening of healthy older adults or those with cognitive concerns was not within the scope of this study and is an understudied topic more generally. Although more research is needed, emerging qualitative research with healthy and cognitively impaired populations can provide a more nuanced examination of patients’, caregivers’, and providers’ attitudes and preferences for cognitive screening [16,71,76,77]. Older adults’ perspectives on cognitive screening are multifaceted and diverse, and a human-centered approach to developing screening paradigms is imperative to prevent stigma and improve early detection [16,77].

### Limitations

Despite its strengths, our study has important limitations, primarily regarding the generalizability of our sample. Before embarking on this project, we knew that the first version of MyCog Mobile would be built for iOS smartphones based on the stipulations of the supporting research grant. Although smartphone ownership is becoming more ubiquitous among older adults, many older adults still do not own smartphones or feel confident using them. Moreover, iPhones are among the most expensive smartphone brands, which may have biased the sample toward higher-income participants. We also sourced participants through User Interviews, a web-based platform that users voluntarily seek out and sign up for, which may have created some self-selection bias in the sample of older adults who participated in individual interviews and prototype testing.

The constraints for the pilot version of MyCog Mobile risk exacerbating disparities in health care access and outcomes for older adults with lower incomes or less technical experience [75-77]. To compensate for the potential selection bias in the recruitment method, we incorporated the views of a diverse panel of community members. To broaden the potential user group of older adults, we intend to develop a MyCog Mobile app for other smartphone operating systems in subsequent versions if the iOS version proves successful. We also plan to conduct additional usability interviews with a subsample of participants from the feasibility and validation study, with special attention to the experiences of older adults with lower income and less experience with technology. Findings from subsequent usability testing will inform further improvements to the user interface before the finalized app is released.

Another important limitation of our sample is that we did not specifically recruit older adults with cognitive impairments. As previously described, clinicians and clinic administrators saw value in reducing the time spent on in-person screenings for most of their patients who are cognitively healthy. Still, clinicians recognized that using MyCog Mobile would be challenging for cognitively impaired older adults and that a portion of older adults with and without cognitive impairment would not complete the MyCog Mobile screener at home. We intend to conduct usability interviews with participants with cognitive impairments during a clinical validation study to understand the accessibility of MyCog Mobile for this population.

Finally, the workflows and designs we proposed are based on individual stakeholders’ self-reported beliefs and behaviors in a hypothetical situation. Usability ratings reflect only the usability of prototype mock-ups tested in the context of a controlled research setting. These ratings, although positive, may not predict actual use in practice [78]. Moreover, as the prototypes were iteratively improved, survey ratings must be considered individually and are descriptive in nature. This study reflects formative research in the context of an ongoing user-centered design and implementation process for MyCog Mobile, and additional feedback from end users is needed to further optimize the user experience and identify additional usability issues [79].

We anticipate unforeseen challenges related to the implementation, compliance, and usability of MyCog Mobile when launched in real-world primary care clinics. Once validated and further refined, the MyCog Mobile app will be piloted in clinics to test its feasibility and continue to optimize the remote cognitive screening process for older adults. We will pay special attention to the technical support needs of older adults as well as additional support that clinics may put in place to increase the at-home screening rate (eg, outreach to increase awareness and caregiver involvement, including MyCog Mobile training videos on their website).

### Conclusions

Through an iterative, human-centered process, we were able to design a viable remote cognitive screening app, MyCog Mobile, and a proposed workflow for primary care settings that was optimized for multiple stakeholders. Insights from clinicians and clinic administrators helped minimize any potential logistical friction related to the implementation of MyCog Mobile as well as maximize its utility in clinical practice. Feedback from older adults helped us design a streamlined user experience that would encourage patients to complete MyCog Mobile at home before a primary care visit. At-home, smartphone-based cognitive screening for primary care settings is a relatively new concept with many potential obstacles as well as benefits. We hope that researchers and app developers interested in cognitive screening can use our design process as a model for future app development, particularly regarding the involvement of multiple stakeholders early and often throughout the iterative design process. This approach can inform not only which features to build but also what not to build to avoid costly mistakes.

This study reflects formative, early-stage user research in the context of an ongoing user-centered design and implementation process for MyCog Mobile. The next steps for MyCog Mobile include validating the tests in clinical and healthy populations and piloting the finalized app in a community primary care clinic. We intend to continue to solicit feedback from stakeholders and refine the app throughout each phase of the project. Once released, MyCog Mobile has the potential to improve primary care clinics’ workflow efficiency, encourage broader routine cognitive screening, and promote serial tracking of cognitive functioning over longer periods. We hope that the direct benefits of MyCog Mobile will subsequently increase the rates of early detection and management of cognitive impairment in older adults.

## Appendix

Multimedia Appendix 1 Dimensional Change Card Sorting activity, version 0 (Mobile Toolbox version).

Multimedia Appendix 2 Picture Sequence Memory activity, version 0 (Mobile Toolbox version).

Multimedia Appendix 3 Dimensional Change Card Sorting activity, version 4 (final MyCog Mobile version).

Multimedia Appendix 4 Picture Sequence Memory activity, version 4 (final MyCog Mobile version).

## Abbreviations

ASQ: After-Scenario Questionnaire
EHR: electronic health record
mHealth: mobile health
PCC: primary care clinician
REDCap: Research Electronic Data Capture
ShARP: Stakeholder-Academic Resource Panel
S-SUS: Simplified System Usability Scale

## Data Availability

Given the small data set, interview, panel, and usability testing data are not publicly available to ensure participants’ privacy. For questions about the data set, please contact the corresponding author.
